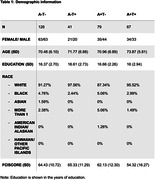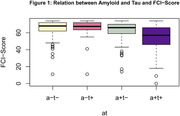# Positive Tau and Amyloid Burden as Potential Predictors for Financial Capacity in Pre‐ Dementia Stages of Alzheimer’s Disease

**DOI:** 10.1002/alz.093256

**Published:** 2025-01-09

**Authors:** Kayla Le, Andrei Bieger, Thomas Hugentobler Schlickmann, Sarah Wehle Gehres, Marco Antônio de Bastiani, Wyllians Vendramini Borelli, Eduardo R. Zimmer, ADNI

**Affiliations:** ^1^ University of Applied Sciences Hamm‐ Lippstadt, Hamm, North Rhine‐ Westphalia Germany; ^2^ Universidade Federal do Rio Grande do Sul, Porto Alegre, Rio Grande do Sul Brazil; ^3^ Federal University of Rio Grande do Sul, Porto Alegre, Rio Grande do Sul Brazil; ^4^ Alzheimer’s Disease Neuroimaging Initiative, http://adni.loni.usc.edu/, CA USA

## Abstract

**Background:**

Amyloid and tau pathologies are the hallmarks of Alzheimer’s disease (AD). Previous research indicated notable connections between financial capacity and AD biomarkers. Here, we aimed to understand whether financial capacity is affected by the cerebral accumulation of tau and amyloid. We hypothesized that amyloid and tau positivity will lead to a lower FCI‐Score.

**Method:**

CU and MCI participants who had completed Financial Capacity Instrument ‐ Short Form (FCI‐SF) assessment, amyloid‐ and tau‐PET scans were selected from the Alzheimer’s Disease Neuroimaging Initiative (ADNI). Linear regression analysis was employed to investigate associations between Amyloid and Tau positivity with FCI‐Score, adjusted for age, gender, education and race (Table 1). Finally, a stepwise regression analysis was conducted to identify the predictors of financial capacity.

**Result:**

Upon conducting a linear regression model on the positive amyloid and tau burden (a+t+), a highly significant negative association between a+t+ and FCI Score was observed (Figure 1). Stepwise regression analysis reiterated these results and both positive amyloid and tau burden (p < 0.01) demonstrated a significant predictive value of the FCI‐Score.

**Conclusion:**

Our results align with previous studies on neurocognitive factors of financial capacity in pre‐ dementia stages, lending additional support for the significant influence of both amyloid and tau positivity in the decline of financial capacity. A decline in financial capacity may indicate pathological buildup and serve as an early warning sign for Alzheimer’s disease. Therefore, future research should continue to investigate the longitudinal relationship between tau, amyloid, financial capacity and other instrumental activities of daily living.